# Inter-anesthesiologist Practice Variation in Vasopressor Administration for Intraoperative Hypotension: A Retrospective Cohort Study

**DOI:** 10.7759/cureus.117418

**Published:** 2026-10-04

**Authors:** Denis George, Nicolas Van Rompaey, Emmanuel Noel

**Affiliations:** 1 Anesthesiology, Centre Hospitalier Universitaire de Tivoli, La Louvière, BEL; 2 Anesthesiology, Centre Hospitalier Interrégional Edith Cavell, Brussels, BEL

**Keywords:** anesthesiologists, case-mix, episode-level analysis, intraoperative hypotension, practice variation, vasopressor administration

## Abstract

Introduction

Intraoperative hypotension (IOH) is common and has been associated with postoperative organ injury, morbidity, and mortality. Although its incidence and treatment thresholds have been investigated, variation in vasopressor administration across individual anesthesiologists remains incompletely characterized. We aimed to assess inter-anesthesiologist variation in documented vasopressor administration in response to IOH during elective non-cardiac surgery.

Methods

We conducted a single-center retrospective cohort study of adults undergoing elective non-cardiac surgery with at least one mean arterial pressure measurement below 65 mmHg. The primary unit of analysis was the hypotensive episode, and the primary outcome was documented ephedrine or phenylephrine administration associated with each episode. Inter-anesthesiologist variation was assessed using multivariable logistic regression adjusted for case-mix and episode order, with procedure-clustered robust covariance. Adjusted probabilities were estimated by marginal standardization.

Results

Among 900 procedures managed by 20 anesthesiologists, 2,146 hypotensive episodes were analyzed, of which 734 (34.2%) were associated with vasopressor administration. After adjustment, anesthesiologist identity remained associated with episode-level vasopressor administration (p<0.001), with marginal standardized probabilities ranging from 3.7% to 52.2%. Findings were consistent across sensitivity analyses.

Conclusions

Substantial inter-anesthesiologist variation in documented vasopressor use was observed after adjustment for measured case-mix variables, highlighting the need to better understand the determinants and clinical significance of provider-associated variation.

## Introduction

Intraoperative hypotension (IOH) is common during anesthesia, although its reported incidence varies widely according to the threshold and duration used to define it [1]. IOH has been associated with postoperative acute kidney injury, myocardial injury, and mortality [2-7]. These associations appear graded, with risk increasing with greater depth and duration of hypotension [7,8]. Taken together, these findings support efforts to limit IOH.

Although intraoperative blood pressure management has been extensively studied, the contribution of individual anesthesiologists to real-world practice variation remains comparatively underexplored. Variation in IOH incidence across anesthesia providers has been reported [9], and prospective work has shown substantial variation in the blood pressure thresholds prompting vasopressor administration [10]. Surveys also suggest heterogeneity in stated treatment thresholds and vasoactive strategies [11]. However, stated practice may not reflect actual decisions made during individual hypotensive episodes, underscoring the need for objective, practice-based measures of variation.

Among the various strategies for managing IOH, vasopressor administration constitutes an identifiable and routinely documented therapeutic intervention, making it particularly well suited to objective assessment of practice variation. We aimed to quantify inter-anesthesiologist variation in documented vasopressor administration for IOH. We hypothesized that the probability of documented vasopressor administration would vary across anesthesiologists after adjustment for measured patient, anesthetic, surgical, and episode-level characteristics.

## Materials and methods

Study design and reporting

We conducted a single-center retrospective observational cohort study at CHU Tivoli, La Louvière, Belgium. The study was approved by the institutional Ethics Committee (accreditation number 096; study protocol number 1356). Because routinely collected anonymized data were used, the requirement for individual informed consent was waived by the Ethics Committee. The manuscript was prepared in accordance with the Strengthening the Reporting of Observational Studies in Epidemiology (STROBE) guidelines [12].

Population

Clinical data for procedures performed from January 1 to March 31, 2019, were extracted locally by the study investigators from the institution's eXacto database (Mexys SA, Chaussée de Binche 101, 7000 Mons, Belgium). No access to or data extraction by the manufacturer was required. The retrospective use of these routinely collected institutional clinical data was covered by the Ethics Committee approval. We included adults undergoing elective non-cardiac surgery under general or neuraxial anesthesia who experienced at least one IOH episode. IOH was defined as a non-invasive mean arterial pressure (MAP) measurement below 65 mmHg. This threshold was selected because an absolute MAP below 65 mmHg is widely used to define clinically relevant IOH and has been associated with postoperative organ injury in observational studies and perioperative consensus recommendations [7,8]. No a priori sample size calculation was performed; all eligible procedures performed during the three-month study period were included.

We excluded cardiac and pediatric surgery, peripheral regional anesthesia without general or neuraxial anesthesia, ASA physical status ≥4, emergency procedures, cesarean delivery, cataract surgery, invasive arterial pressure monitoring, and procedures requiring continuous vasoactive drug infusion. One anesthesiologist contributed only two eligible procedures (four hypotensive episodes, none with documented study vasopressor administration) and was excluded from the primary adjusted provider-specific analysis because this sparse stratum yielded an unstable provider estimate. Procedures involving invasive arterial pressure monitoring were excluded to focus the analysis on routine elective non-cardiac surgical practice and to limit the inclusion of higher-risk patients and procedures requiring more intensive hemodynamic monitoring.

Blood pressure measurements and hypotensive episodes

Non-invasive oscillometric blood pressure measurements were electronically recorded. Measurement frequency was determined by the treating anesthesiologist during routine practice, most commonly every 2.5 or 5 minutes. A hypotensive episode began with the first MAP measurement below 65 mmHg and continued as long as successive MAP measurements remained below this threshold. The first subsequent MAP measurement ≥65 mmHg marked the end of the episode. A new MAP measurement below 65 mmHg occurring after at least one measurement ≥65 mmHg was considered a new episode. The source database recorded the onset and assigned duration of each episode; across the cohort, the number of episodes per procedure ranged up to 14, reflecting the observed distribution rather than a system-imposed limit. Each episode was treated as a separate observation while retaining the procedure identifier to account for repeated episodes within the same procedure.

Outcome and exposure

The primary outcome was a binary episode-level indicator of documented ephedrine or phenylephrine administration associated with each hypotensive episode in the extracted episode-level database. Vasopressor administrations were manually entered into the electronic anesthesia record by the anesthesiologist responsible for the patient and were assigned to a hypotensive episode when the recorded administration time fell within that episode. Ephedrine and phenylephrine were the only vasopressors routinely administered as boluses at CHU Tivoli during the study period. Norepinephrine was used as a continuous infusion rather than as a bolus; procedures requiring continuous vasoactive drug infusion were excluded from the study. The primary exposure was the identity of the anesthesiologist recorded for the procedure. Episodes were therefore described as with or without documented study vasopressor administration rather than as treated or untreated hypotension.

Covariates

Covariates were prespecified on clinical grounds and availability: age, sex, ASA physical status, hypertension, cardiac disease, beta-blocker, calcium-channel blocker, angiotensin-converting enzyme inhibitor, angiotensin receptor blocker, diuretic, centrally acting antihypertensive, general versus neuraxial anesthesia, anesthesia duration, laparoscopy, sitting position, surgical category, and episode order. Episode order was grouped as first, second, third, or fourth and subsequent episode. Surgical categories were digestive/general/bariatric surgery, vascular surgery, gastrointestinal endoscopy, gynecology, maxillofacial/dental surgery, neurosurgery/spine surgery, otorhinolaryngology/head and neck surgery, orthopedics/trauma, breast/plastic/cutaneous surgery, urology, and other specialties. Body mass index was added only in a complete-case sensitivity analysis. Episode duration and the total number of episodes were not used as predictors because vasopressor administration could modify them.

Statistical analysis

Procedure-level characteristics are summarized as median (interquartile range) or number (percentage), with two-sided p values <0.05 considered statistically significant. The primary analysis used episode-level logistic regression fitted with stats::glm(), with anesthesiologist identity modeled as a categorical fixed effect and all prespecified covariates entered simultaneously. Age and anesthesia duration were modeled as continuous predictors assuming linear relationships on the logit scale; departures from linearity were not formally assessed. Body mass index was the only covariate with missing data and was therefore excluded from the primary model and evaluated in a complete-case sensitivity analysis. Within-procedure correlation among repeated hypotensive episodes was addressed using procedure-clustered robust sandwich covariance estimates obtained with sandwich::vcovCL() using type = "HC1" and cadjust = TRUE, applying an HC1 degrees-of-freedom correction and a finite-cluster adjustment. The overall anesthesiologist effect was assessed using a cluster-robust global Wald test of the 19 anesthesiologist coefficients, with one anesthesiologist as the reference category.

Adjusted anesthesiologist-specific probabilities of vasopressor administration were estimated by marginal standardization using the marginaleffects package, averaging model-based predictions over the observed covariate distribution. Confidence intervals were obtained by the delta method on the logit scale using the procedure-clustered robust covariance matrix and were back-transformed to the probability scale. For descriptive purposes, overall model discrimination was summarized by the area under the receiver operating characteristic curve (AUC); prediction was not an objective of the study.

Sensitivity analyses included four alternative specifications. Restricting the cohort to anesthesiologists with at least 20 procedures used the same episode-level model and a procedure-clustered robust global Wald test. The ≥20-procedure threshold was selected pragmatically to reduce the influence of sparsely represented provider strata and unstable provider-specific estimates while retaining most anesthesiologists in the analysis. The first-episode-only analysis included one independent episode per procedure, retained the same prespecified covariates as the primary model except for episode order, which was constant by design, and used a global likelihood-ratio test comparing the corresponding adjusted models with and without anesthesiologist identity. The procedure-level analysis defined vasopressor administration as a binary outcome at the procedure level and likewise used a global likelihood-ratio test comparing the corresponding adjusted models with and without anesthesiologist identity. The complete-case analysis additionally adjusting for body mass index used the primary episode-level model with procedure-clustered robust covariance and a global Wald test. As an additional robustness analysis, the primary model was refitted without the six antihypertensive medication classes, and the anesthesiologist association was assessed using the same procedure-clustered robust global Wald test.

As a post hoc exploratory analysis, we additionally examined vasopressor agent choice (ephedrine versus phenylephrine) among episodes with documented study vasopressor administration. Episodes in which both agents were documented were excluded from the single-agent comparison. Because this analysis was not prespecified and relevant hemodynamic determinants of agent selection were unavailable, agent choice was compared across anesthesiologists using an unadjusted Pearson chi-square test, restricted to anesthesiologists with at least 20 single-agent episodes to reduce instability from sparse provider strata. As a sensitivity analysis, agent choice was also compared using only the first single-agent episode of each procedure, with statistical significance confirmed using a Monte Carlo exact test.

All analyses were performed using R software (version 3.6.2; R Core Team, 2019).

## Results

Study population and episodes

Of 3,825 anesthetic procedures performed during the study period, 2,633 remained after initial database exclusions and 1,176 had at least one MAP measurement below 65 mmHg. After detailed eligibility review, 902 procedures containing 2,150 hypotensive episodes from 21 anesthesiologists were eligible. One anesthesiologist contributed only two procedures and four episodes, none with documented study vasopressor administration, and was excluded from the adjusted provider-specific analysis. The primary cohort therefore comprised 900 procedures, 2,146 hypotensive episodes, and 20 anesthesiologists. A study vasopressor was documented for 734 episodes (34.2%): ephedrine alone in 565 (77.0%), phenylephrine alone in 151 (20.6%), and both agents in 18 (2.5%). The flow of procedures from initial eligibility screening to the primary adjusted analysis is summarized in Figure 1.

**Figure 1:**
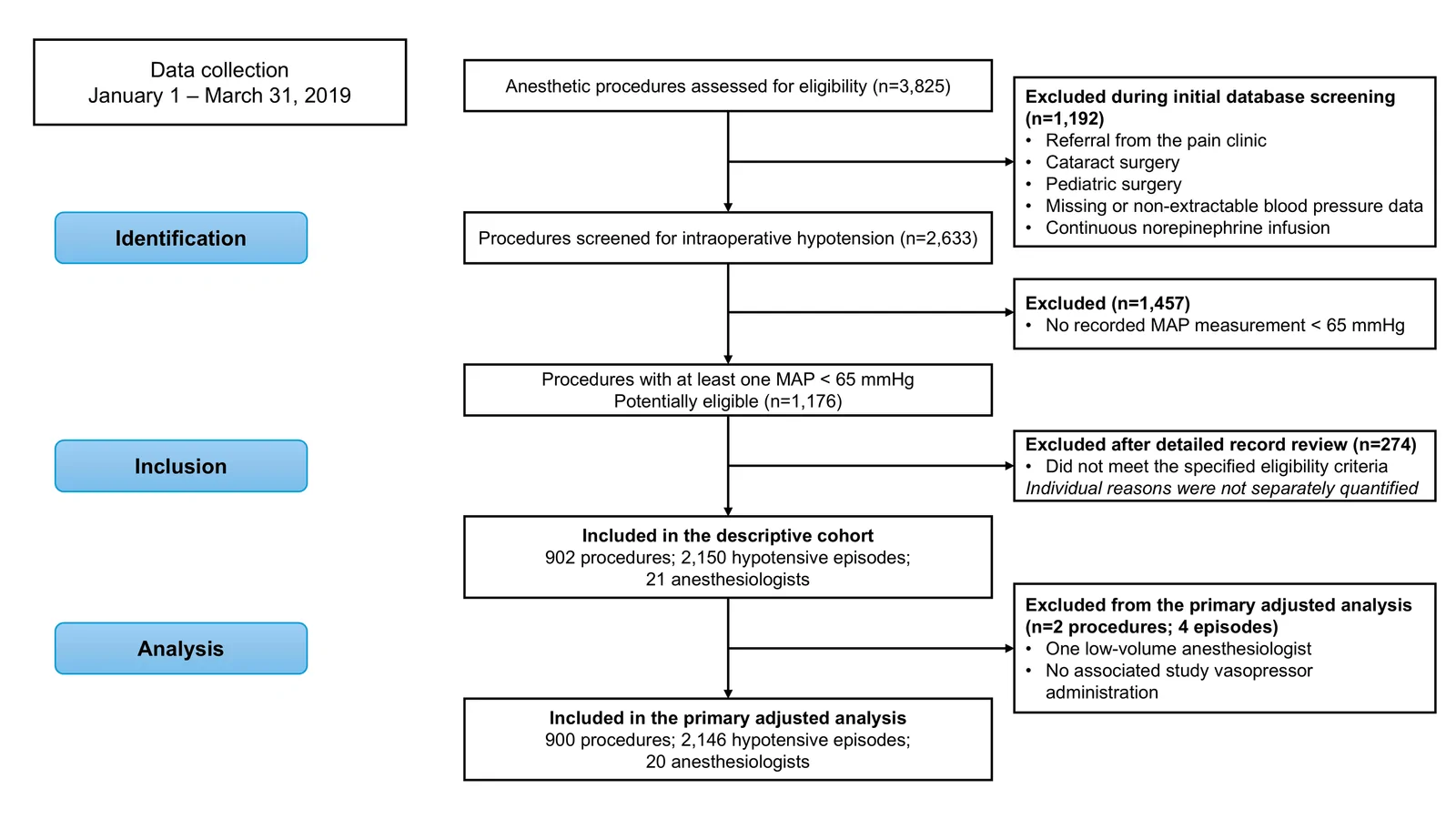
STROBE flow diagram of procedure selection from initial eligibility screening to the primary adjusted analysis. MAP, Mean Arterial Pressure; STROBE, Strengthening the Reporting of Observational Studies in Epidemiology

Procedure-level characteristics are presented in Table 1. Median age was 55 (41-66) years, 332 procedures (36.9%) involved a patient with hypertension, and 320 (35.6%) involved at least one antihypertensive medication class. The median anesthesia duration was 80.5 (47.3-119.0) minutes.

**Table 1:** Procedure-level characteristics of the primary analysis cohort. Values are median (interquartile range) or n (%). ASA, American Society of Anesthesiologists; ACE, Angiotensin-Converting Enzyme.

Characteristic	Overall (n=900)
Age, years	55 (41-66)
Male sex	387 (43.0%)
Body mass index, kg/m^2^	26.5 (23.1-31.2); n=873
ASA physical status 1/2/3	266 (29.6%)/ 513 (57.0%)/121 (13.4%)
Hypertension	332 (36.9%)
Cardiac disease	67 (7.4%)
Any antihypertensive medication	320 (35.6%)
Beta-blocker	185 (20.6%)
Calcium-channel blocker	103 (11.4%)
ACE inhibitor	107 (11.9%)
Angiotensin receptor blocker	83 (9.2%)
Diuretic	103 (11.4%)
Centrally acting antihypertensive	15 (1.7%)
Neuraxial anesthesia	53 (5.9%)
Anesthesia duration, min	80.5 (47.3-119.0)
Laparoscopy	203 (22.6%)
Sitting position	23 (2.6%)

Provider-level procedural volume, number of hypotensive episodes, and crude vasopressor administration rates are presented in Table 2.

**Table 2:** Provider-level procedural volume, hypotensive episodes, and crude vasopressor administration. Crude percentages represent the proportion of hypotensive episodes with documented ephedrine or phenylephrine administration for each anesthesiologist.

Anesthesiologist	Procedures, n	Hypotensive episodes, n	Episodes with documented study vasopressor, n (%)
A01	79	202	29 (14.4)
A02	18	27	1 (3.7)
A03	26	57	15 (26.3)
A04	27	53	18 (34.0)
A05	10	16	4 (25.0)
A06	27	63	12 (19.0)
A07	33	80	14 (17.5)
A08	51	96	33 (34.4)
A09	61	171	49 (28.7)
A10	20	31	3 (9.7)
A11	70	182	47 (25.8)
A12	25	52	16 (30.8)
A13	31	70	16 (22.9)
A14	32	77	15 (19.5)
A15	49	89	39 (43.8)
A16	83	174	50 (28.7)
A17	57	169	91 (53.8)
A18	57	151	74 (49.0)
A19	79	191	105 (55.0)
A20	65	195	103 (52.8)
Total	900	2,146	734 (34.2)

Primary episode-level outcome

After adjustment for the prespecified case-mix, antihypertensive medication classes, and episode order, anesthesiologist identity remained globally associated with documented vasopressor administration (procedure-clustered robust Wald chi-square=128.3, 19 degrees of freedom; p<0.001). The AUC of the complete model was 0.785. Marginal standardized probabilities ranged from 3.7% to 52.2% across anesthesiologists (Figure 2). Confidence intervals were wider for anesthesiologists contributing fewer episodes.

**Figure 2:**
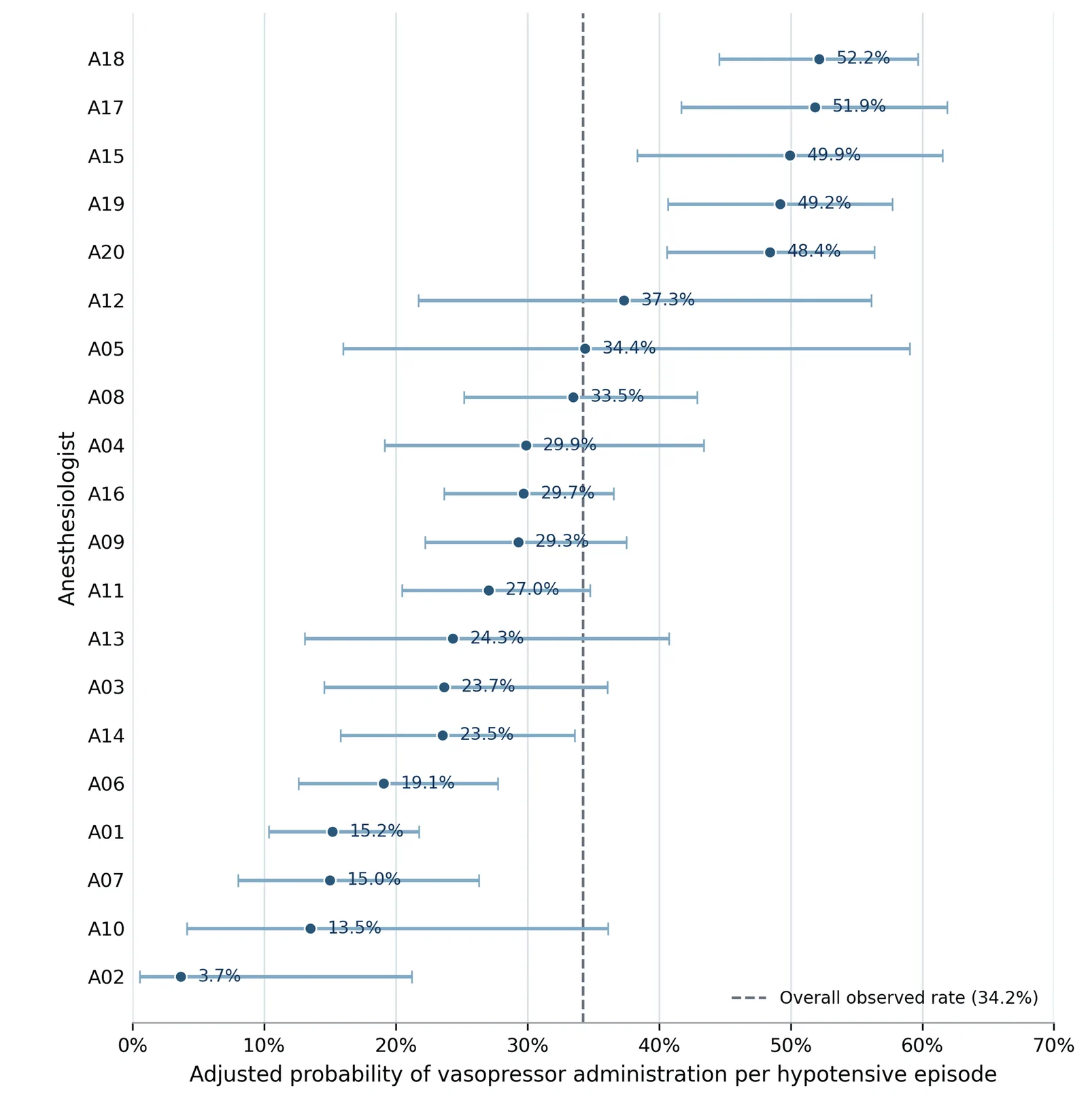
Adjusted probability of documented vasopressor administration for a hypotensive episode according to the anesthesiologist. Points represent marginal standardized probabilities and horizontal bars represent their 95% confidence intervals. The dashed line indicates the overall observed episode-level proportion. Estimates were adjusted for age, sex, ASA class, hypertension, six antihypertensive medication classes, cardiac disease, anesthesia type and duration, laparoscopy, sitting position, surgical category, and episode order, with robust covariance clustered by procedure.

Selected case-mix associations are shown in Table 3. Older age and sitting position were associated with a higher probability of documented vasopressor administration, whereas laparoscopy and fourth or subsequent hypotensive episodes were associated with a lower probability. The estimate for centrally acting antihypertensive medication was elevated, but only 15 procedures involved this medication class and the finding should be considered exploratory. Full model estimates are shown in Table 3.

**Table 3:** Case-mix-adjusted episode-level logistic regression for documented vasopressor administration. The global p-value for anesthesiologist identity was <0.001. The reference surgical category was digestive/general/bariatric surgery. Individual anesthesiologist coefficients are not shown because they depend on the arbitrary reference anesthesiologist; standardized probabilities are presented in Figure 2. ASA, American Society of Anesthesiologists; ACE, Angiotensin-Converting Enzyme.

Variable	Adjusted OR	95% CI	p-value
Age, per 10 years	1.38	1.25-1.52	<0.001
Male sex	1.04	0.80-1.35	0.788
ASA: 2 vs 1	0.84	0.60-1.18	0.311
ASA: 3 vs 1	1	0.61-1.63	0.986
Hypertension	1.40	0.88-2.24	0.159
Beta-blocker	0.94	0.65-1.36	0.744
Calcium-channel blocker	1.07	0.71-1.61	0.748
ACE inhibitor	1.27	0.81-1.99	0.295
Angiotensin receptor blocker	0.93	0.61-1.42	0.752
Diuretic	0.74	0.49-1.11	0.145
Centrally acting antihypertensive	2.56	1.22-5.35	0.013
Cardiac disease	0.91	0.55-1.50	0.713
Neuraxial anesthesia	2.07	0.92-4.66	0.079
Anesthesia duration, per 30 min	1.05	1.00-1.10	0.051
Laparoscopy	0.56	0.32-0.98	0.041
Sitting position	3	1.32-6.86	0.009
Surgery: Other specialties vs digestive/general/bariatric	1.34	0.53-3.41	0.537
Surgery: Vascular surgery vs digestive/general/bariatric	0.5	0.26-0.98	0.043
Surgery: Gastrointestinal endoscopy vs digestive/general/bariatric	0.36	0.16-0.82	0.015
Surgery: Gynecology vs digestive/general/bariatric	0.46	0.25-0.85	0.013
Surgery: Maxillofacial/dental surgery vs digestive/general/bariatric	0.28	0.12-0.66	0.004
Surgery: Neurosurgery/spine surgery vs digestive/general/bariatric	1.01	0.53-1.91	0.986
Surgery: ENT/head and neck surgery vs digestive/general/bariatric	0.35	0.18-0.71	0.003
Surgery: Orthopedics/trauma vs digestive/general/bariatric	0.51	0.27-0.96	0.036
Surgery: Breast/plastic/cutaneous surgery vs digestive/general/bariatric	0.45	0.22-0.91	0.026
Surgery: Urology vs digestive/general/bariatric	0.42	0.17-1.02	0.055
Episode order: 2 vs 1	1.05	0.81-1.36	0.701
Episode order: 3 vs 1	0.87	0.63-1.20	0.411
Episode order: 4+ vs 1	0.48	0.34-0.69	<0.001

Exploratory analysis of vasopressor agent choice

Restricting the analysis to the 716 episodes in which a single study vasopressor was documented, phenylephrine accounted for 151 (21.1%) administrations and ephedrine for 565 (78.9%). Among the 606 single-agent episodes contributed by the 10 anesthesiologists with at least 20 such episodes, vasopressor choice differed across anesthesiologists (Pearson chi-square=69.3, 9 degrees of freedom; p<0.001; Cramér's V=0.34), with phenylephrine use ranging from 0.0% to 39.4% (Table 4). This pattern remained significant when the analysis was restricted to the first single-agent episode of each procedure (334 ephedrine and 70 phenylephrine administrations among 404 procedures; chi-square=68.5, 19 degrees of freedom; p<0.001; Monte Carlo exact p<0.001). This analysis was post-hoc, exploratory, and unadjusted for case-mix.

**Table 4:** Vasopressor agent choice by anesthesiologist among episodes with a single documented study vasopressor, restricted to anesthesiologists with at least 20 such episodes. This post-hoc, exploratory, unadjusted analysis excludes 18 episodes in which both ephedrine and phenylephrine were documented and 110 single-agent episodes contributed by anesthesiologists with fewer than 20 such episodes, whose estimates would be unstable. Pearson chi-square=69.3, 9 degrees of freedom, p<0.001; Cramér's V=0.34.

Anesthesiologist	Single-agent vasopressor episodes	Ephedrine	Phenylephrine	Phenylephrine, %
A16	50	50	0	0.00%
A18	71	68	3	4.20%
A20	103	97	6	5.80%
A11	45	36	9	20.00%
A15	38	30	8	21.10%
A01	29	22	7	24.10%
A19	98	69	29	29.60%
A09	48	32	16	33.30%
A17	91	58	33	36.30%
A08	33	20	13	39.40%

Sensitivity analyses

The global anesthesiologist association remained statistically significant in every prespecified sensitivity analysis (Table 5). Adjustment for the six antihypertensive medication classes had virtually no effect on the global anesthesiologist association: the robust Wald chi-square was 128.3 in the fully adjusted model and 128.2 in the otherwise identical model excluding these medications.

**Table 5:** Sensitivity analyses of the anesthesiologist association. Global tests were procedure-clustered robust Wald tests for the “Providers with ≥20 procedures” and “Episode-level plus BMI, complete cases” analyses, and likelihood-ratio tests comparing the corresponding adjusted models with and without anesthesiologist identity for the “First episode only” and procedure-level analyses.

Analysis	N	Procedures	Events	Providers	Global test	p-value
Providers with ≥20 procedures	2103	872	729	18	120.2 (17 df)	<0.001
First episode only	900	900	301	20	63.9 (19 df)	<0.001
Procedure-level: any episode with vasopressor administration	900	900	412	20	92.3 (19 df)	<0.001
Episode-level plus BMI, complete cases	2095	873	723	20	122.1 (19 df)	<0.001

## Discussion

In this retrospective cohort of 900 elective non-cardiac procedures and 2,146 hypotensive episodes, documented ephedrine or phenylephrine administration varied substantially across anesthesiologists. The association persisted after adjustment for measured patient characteristics, antihypertensive medication classes, anesthetic and surgical characteristics, and episode order. Standardized episode-level probabilities ranged from approximately 4% to 52%. These findings characterize provider-associated variation in documented vasopressor use rather than variation in overall IOH management.

The episode-level approach improves alignment between the clinical decision and the analytical unit. A procedure may contain several hypotensive episodes, only some of which are associated with vasopressor administration; a procedure-level outcome therefore obscures within-procedure heterogeneity. Conversely, treating repeated episodes as independent would overstate precision. We therefore evaluated each episode while using procedure-clustered robust covariance to account for repeated observations.

Our results complement previous studies examining provider-associated blood pressure patterns and treatment thresholds. Saasouh et al. described variability in IOH incidence across community anesthesia providers [9], and Wickham et al. found substantial variation between intended and applied blood pressure thresholds prompting vasopressor administration [10]. Survey data have similarly suggested heterogeneity in anesthesiologists' stated treatment thresholds and vasoactive strategies [11], but self-reported preferences may not capture what actually occurs at the bedside. The present study extends this literature by using documented administrations linked to individually identified hypotensive episodes and asks whether vasopressor use varies across anesthesiologists once a hypotensive episode has been identified.

The post hoc analysis of vasopressor agent choice suggests that inter-anesthesiologist variation may operate at two distinct levels: whether a vasopressor is documented for a hypotensive episode and which agent is selected when a vasopressor is used. Vasopressor selection may depend on the underlying hemodynamic context and the presumed mechanism of hypotension [13]. Because heart rate, presumed cardiac output, the mechanism of hypotension, and anesthetic depth were unavailable, this exploratory analysis could not account for clinically legitimate reasons for agent selection and should be interpreted as hypothesis-generating rather than confirmatory. Accordingly, the observed inter-anesthesiologist differences in agent choice should not be interpreted as reflecting provider preference alone.

Several case-mix covariates showed plausible associations with documented vasopressor use, but these coefficients were included primarily for adjustment rather than as study endpoints. Given the number of covariates examined and the limited information on episode physiology, individual case-mix associations should be considered hypothesis-generating. Adjustment for antihypertensive medication use did not attenuate the inter-anesthesiologist association, suggesting that differences in these medications did not explain the observed practice variation.

The lower probability of vasopressor administration during fourth and subsequent episodes may reflect clinical adaptation, repeated brief threshold crossings, or changes in anesthetic or fluid management. Because MAP nadir, heart rate, anesthetic adjustments, fluid boluses, and the physiological mechanism of hypotension were unavailable, this association cannot be assigned a single explanation.

The magnitude of the adjusted provider-associated variation may have clinically relevant implications. Although our study was not designed to define an optimal treatment threshold or vasopressor administration rate, substantial provider-associated variation persisted after adjustment for measured case-mix variables. Future studies could evaluate whether episode-level risk-adjusted audit and feedback, together with improved documentation of hypotension severity, may help characterize the determinants and clinical significance of provider-associated variation [14]. Such studies should also determine whether this variation is associated with differences in hypotension burden and postoperative outcomes.

Strengths and limitations

Strengths include the clinically aligned episode-level outcome, analysis of 20 individual anesthesiologists, adjustment for prespecified case-mix variables, explicit handling of repeated episodes within procedures, marginal standardized probabilities with confidence intervals, and several sensitivity analyses. The analysis also avoided adjusting for episode duration or total episode count, which could themselves be modified by treatment.

The study has important limitations. First, only documented ephedrine and phenylephrine administrations were captured. Fluid administration, reduction of anesthetic depth, changes in position, observation of self-limited episodes, and other vasoactive drugs were not reliably available. Absence of a study vasopressor therefore cannot be interpreted as absence of IOH treatment. Second, episode severity was incompletely characterized: MAP nadir, area under 65 mmHg, baseline-relative reduction, heart rate, and mechanism were unavailable. These unavailable episode-level severity measures may have differed across anesthesiologists and could therefore contribute to the observed provider-associated variation; residual confounding by indication and severity is consequently likely. Third, episode identification depended on intermittent non-invasive measurements obtained at variable intervals. Differences in measurement frequency could therefore have influenced the detection, apparent duration, and number of hypotensive episodes and represent a potential source of measurement heterogeneity. Fourth, vasopressor administrations were manually entered by the treating anesthesiologist. Incomplete or inaccurate documentation may therefore have resulted in misclassification of episode-level vasopressor administration, and systematic differences in documentation practices across anesthesiologists could have contributed to the observed provider-associated variation. Fifth, anesthesiologist assignment was not randomized and may proxy unmeasured case-mix, scheduling, supervision, or documentation. Sixth, fixed effects quantify association among the anesthesiologists observed in this center but do not estimate a generalizable between-provider variance. Seventh, the exploratory analysis of vasopressor agent choice was post hoc and unadjusted and could not account for hemodynamic determinants of agent selection. Finally, this was a single-center cohort conducted over a three-month period. Practice patterns, staffing, and case mix may differ across institutions and over time, limiting the generalizability of the observed provider-associated variation.

## Conclusions

Documented vasopressor use for IOH varied substantially across anesthesiologists after adjustment for measured case-mix, with consistent findings across sensitivity analyses. This variation highlights the need to better understand the determinants and clinical significance of provider-associated variation. Because hypotension severity and non-vasopressor interventions were unavailable, these findings reflect variation in documented vasopressor use rather than overall IOH management.
